# Ages of hepatocellular carcinoma occurrence and life expectancy are associated with a UGT2B28 genomic variation

**DOI:** 10.1186/s12885-019-6409-3

**Published:** 2019-12-05

**Authors:** Puo-Hsien Le, Chia-Jung Kuo, Yi-Chung Hsieh, Tsung-Hsing Chen, Chih-Lang Lin, Chau-Ting Yeh, Kung-Hao Liang

**Affiliations:** 1 Liver Research Center, Linkou Chang Gung Memorial Hospital, 5, Fu-Shin street, Kuei-Shan District, Taoyuan, Taiwan; 2 Department of Gastroenterology and Hepatology, Linkou Chang Gung Memorial Hospital, Taoyuan, Taiwan; 3 Liver Research Unit, Keelung Chang Gung Memorial Hospital, Keelung, Taiwan; 4 College of Medicine, Chang Gung University, Taoyuan, Taiwan; 5 Department of Medical Research, Taipei Veterans General Hospital, Taipei, Taiwan; 6 Institute of Food Safety and Health Risk Assessment, National Yang-Ming University, Taipei, Taiwan; 7 Institute of Biomedical Informatics, National Yang-Ming University, Taipei, Taiwan

**Keywords:** Young hepatocellular carcinoma; age of death, Xenobiotic metabolizing enzymes, Alcoholism

## Abstract

**Background:**

Hepatocellular carcinoma (HCC) is an aggressive solid tumor. HCC occurred at younger and elder ages were considered driven by different oncogenic mechanisms, and they demonstrated distinct clinical courses.

**Methods:**

A total of 382 HCC patients treated by surgical resections was analyzed.

**Results:**

A univariate-multivariate analysis showed that viral etiology (chronic hepatitis B, C) and the UDP glucuronosyltransferase family 2 member B28 (*UGT2B28*) genomic variant rs2132039 were independently associated with the age at presentation of HCC (all adjusted *P* < 0.05). An extensive evaluations of clinicalpathological factors showed that the age (Odds ratio [OR], 1.016; 95% confidence interval [CI], 1.001–1.032; adjusted *P* = 0.037) and ascites (OR, 3.505; CI, 1.358–9.048; adjusted *P* = 0.010) were two independent factors associated with this genomic variant. The age was 54.1 ± 14.6 years for patients with the “TT” variant type, and 58.2 ± 13.7 years for those with the “Non-TT” variant type. The age disparity was most prominent in alcoholic patients (OR, 1.079; CI, 1.035–1.125; *P* < 0.001, age of “TT”, 49.6 ± 12.2; age of “non-TT”, 59.3 ± 10.7). This genomic variant was also associated with age of recurrence (*P* = 0.025), distant metastasis (*P* = 0.024) and HCC-related death (*P* = 0.008) in non-censored patients.

**Conclusions:**

An *UGT2B28* genomic variant was indicative of the age of HCC presentation, recurrence, distant metastasis and death.

## Background

Hepatocellular carcinoma (HCC) is a prevalent malignancy with an age-standardized rates of 10.1 per 100,000 person-years in the world [1]. Patients diagnosed as early stages of HCC can be treated by curative methods such as surgical resection and liver transplantation [2, 3]. In contrast, patients in advanced stages were either due to delayed diagnosis, or failure of previous treatments. They can only be treated by palliative methods [2, 3]. Early hepatocellular carcinoma (HCC) often lacks overt clinical symptoms [4], therefore, susceptible patients need to schedule regular surveillance ahead of time. Ultrasound and the alpha-fetoprotein levels are important surveillance tools which have demonstrated their effectiveness in enabling early HCC detection and in increasing life expectancy [3, 5–8]. However, the ages of HCC presentation is not homogeneous in different patient subgroups. Therefore, biomarkers indicating the starting ages of surveillance would have great clinical values. HCC occurred at younger and elder ages have been thought to have distinct oncogenic mechanisms [9] and possibly subsequent clinical course [10]. It was currently unknown how the age at presentation was related to genetics, despite the observation that different ethnic groups have different age of presentation [3].

Alcoholism, as well as the hepatitis B and C viral infections (HBV and HCV), are the major etiologies of HCC [11–19]. Treatments of viral infections have improve significantly in recent years. In chronic hepatitis B patients, the risk of HCC is positively related to serum HBV DNA levels [11, 20], and both of them can be substantially reduced by anti-HBV therapy [21, 22]. Besides, nationwide hepatitis B vaccination program has reduced HBV carrier rate significantly in Taiwan, a former HBV endemic region [23]. HCV infection is also one important etiology of HCC particularly in western countries [24]. Direct-acting antivirals can achieve 100% sustained virologic response rate [25–28], thereby reducing the risk of HCC [29]. As a result, alcohol-related HCC are more and more important, due to the reduction of relative importance of other etiologies.

The *UDP glucuronosyltransferase family 2 member B28 (UGT2B28)* gene encodes an important xenobiotic metabolizing enzyme abundantly expressed in the human liver and kidney, and are responsible for the metabolisms of bile acids and sex hormones [30–33]. The copy number variations of *UGT2B28* are associated with the risks of prostate cancers, esophageal squamous cell cancers, and colorectal cancers [34–37]. The genomic variant *UGT2B28*-rs2132039 and an adjacent copy number variation CNP605 have recently been reported to be associated with the natural history of chronic hepatitis B, particularly the e-antigen seroconversion [38]. The two adjacent genomic variants were surrogates of each other [38]. However, the role of *UGT2B28* in the clinical course of liver diseases, particularly the occurrence of HCC, has not been studied to date. Thus, we investigated the relationship between *UGT2B28*-rs2132039 genomic variant and the clinicopathological features, particularly the age at presentation in early HCC patients.

## Methods

### Patients

**Fig. 1:**
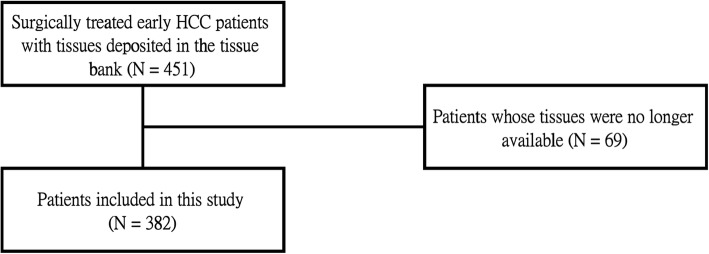
The flowchart of patient enrollment in this study

A screening in the tissue bank of the Chang Gung Memorial Hospital, Linko, Taiwan, identified 451 early HCC patients who have their surgical tissues deposited. Among them, the tissues of 69 patients were no longer available. The remaining 382 HCC patients were then included in this study (Fig. 1). Most tumor characteristics of patients included and not included in this study were similar, except tumor sizes (Additional file 4: Table S1). After the genomic variants were detected from the non-tumor part of the 382 surgical tissues, relevant clinical data were retrospectively retrieved from the clinical charts, including the ages at the diagnosis of HCC, gender, liver cirrhosis, ascites, alcoholism (defined by an average alcohol consumption > 210 g per week in males or > 140 per week in females over at least a 2-year period with physical or psychological dependence), HBV surface antigen (HBsAg), antibody to HCV (anti-HCV), prothrombin time (PT), aspartate transaminase (AST), alanine transaminase (ALT), bilirubin, albumin, creatinine, α-fetoprotein (AFP), tumor size, tumor number, capsule, tumor grade, macrovascular invasion and microvascular invasion. Patients with both HBsAg positivity and antibody anti-HCV positivity were considered as co-infection. We also analyzed the subsequent clinical events after the surgery, including local recurrence, distant metastasis and death, for a follow-up period of 49.2 ± 30.8 months.

### Genotyping of genomic variant

Fresh-frozen surgical tissues were thawed, and then the total DNA was extracted from the non-tumor part of these samples. The polymerase chain reaction method was then used for amplifying the DNA carrying the UDP glucuronosyltransferase family 2 member B28 (UGT2B28) rs2132039 genomic variant using the primers 5′-GAGGCTCCATCATAGTCTGGC-3′ and 5′-TTGCCTGGCTTCTCATTGTT-3′. The amplicon sequence was shown in Additional file 1: Figure S1. Conventional Sanger sequencing was then performed, and the base-calling was done on the sequencing trace files using the public-domain novoSNP bioinformatics tool [39]. Ambiguous calls which cannot be classified by the software were called by human curators.

### Statistical analysis

Categorical variables were summarized as absolute numbers and percentages, and compared using the Chi-squared test. Continuous variables were summarized as median and range, and compared using Mann-Whitney test, or two-sample t-test with unequal variance. Univariate and multivariate analyses were performed by either linear, logistic or Cox regression. The results were shown as odd ratios (OR), hazard ratios (HR), 95% confidence interval (CI) and *P* values. The results were considered to indicate a statistically significant difference when P was less than 0.05. All statistical calculations were performed using SPSS software, version 21 (IBM, Armonk, NY, USA).

## Results

### Evaluating clinical factors associated with age of HCC presentation

Table 1 summarizes the clinical variables of the patients included. The median age at HCC diagnosis was 58 years. The male to female ratio was 3.39. A total of 181 (47.4%) patients had the rs2132039-“TT” variant type, while 201 (52.6%) patients had the “Non-TT” variant type **(**Table 1**)**. We first asked which clinical factors were associated with the age of HCC presentation. It was found that viral etiology (chronic hepatitis B, C) and the *UGT2B28* rs2132039 genomic variant were independently associated with the age (all adjusted *P* < 0.05, Table 2).

**Table 1:** Baseline characteristics of 382 HCC patients in this study

Characteristic	Values
Gender, male, *n* (%)	295 (77.2%)
Age at diagnosis, years, median (range)	58.0 (19.0–87.0)
Tumor number, median (range)	1.0 (1.0–10.0)
Capsule, *n* (%)	280 (73.3%)
Tumor grade, median (range)	3.0 (1.0–4.0)
Macrovascular invasion, *n* (%)	41 (10.7%)
Microvascular invasion, *n* (%)	121 (31.7%)
Tumor size, cm, median (range)	4.3 (0.7–20.0)
Cirrhosis, *n* (%)	225 (58.9%)
Ascites, n (%)	26 (6.8%)
HBV alone, *n* (%)	228 (59.7%)
HCV alone, *n* (%)	63 (16.5%)
HBV + HCV, *n* (%)	36 (9.4%)
NBNC, n (%)	55 (14.4%)
Alcoholism (%)	97 (25.4%)
UGT2B28 rs2132039 TT, *n* (%)	181 (47.4%)
UGT2B28 rs2132039 Non-TT, *n* (%)	201 (52.6%)
Prothrombin time, second, median (rang)	11.9 (9.0–19.5)
AST, U/L, median (range)	37.0 (11.0–559.0)
ALT, U/L, median (range)	39.0 (7.0–749.0)
Bilirubin, mg/dL, median (range)	0.8 (0.3–15.3)
Albumin, g/dL, median (range)	4.1 (1.7–5.1)
Creatinine, mg/dL, median (range)	1.0 (0.4–15.4)
Alpha-fetoprotein, ng/mL, median (range)	26.5 (1.0–685,353.0)
Recurrent, *n* (%)	212 (55.5%)
Recurrent time, month, median (range)	12.9 (1.0–114.7)
Metastasis, *n* (%)	73 (19.1%)
Metastatic time, month, median (range)	13.0 (1.0–99.2)
Death, *n* (%)	47 (12.3%)
Survival time, month, median (range)	22.5 (0.1–73.7)

*Abbreviations*: *HBV* Hepatitis B virus carrier, *HCV* Hepatitis C virus carrier, *HBV + HCV* Co-infection of hepatitis B virus and hepatitis C virus, *NBNC* Non-hepatitis B/hepatitis C virus carrier, *AST* Aspartate aminotransferase, *ALT* Alanine aminotransferase

**Table 2:** Univariate and multivariate linear regression analysis of clinical factors associated to the age at diagnosis

	Univariate Analysis				Multivariate Analysis			
	slope	CI-low	CI-high	*P*	slope	CI-low	CI-high	*P*
Gender, male	−2.468	−5.884	0.947	0.156				
Etiology								
B	−10.083	−13.02	−7.146	< 0.001*	−7.292	−10.453	−4.132	< 0.001*
C	10.061	6.944	13.177	< 0.001*	6.415	3.064	9.766	< 0.001*
Alcoholism	−2.482	−5.772	0.808	0.139				
Cirrhosis	1.149	−1.768	4.065	0.439				
Ascites	0.193	−5.51	5.895	0.947				
AST, U/L	−0.014	− 0.036	0.007	0.194				
ALT, U/L	−0.016	− 0.034	0.002	0.089				
Tumor size, cm	−0.240	− 0.602	0.122	0.193				
UGT2B28 rs2132039								
TT	−4.106	−6.952	−1.26	0.005*	−2.842	−5.52	−0.164	0.038*

*P < 0.05

The fact that the genomic variant was associated with the age, independent of viral etiology, was particularly interesting. Therefore, we examined an extensive list of clinicalpathological variables for their associations with the genomic variant. The “TT” and “non-TT” counts does not have significant difference between cirrhotic and non-cirrhotic patients (*P* = 0.307). The univariate logistic regression analysis showed that age (OR 1.021, 95% C.I. 1.006–1.036, *P* = 0.005), ascites (OR 3.223, 95% C.I. 1.264–8.215, *P* = 0.014) and hepatitis C infection (OR 1.839, 95% C.I. 1.148–2.945, *P* = 0.011) were associated with the genomic variant **(**Table 3**)**. Multivariate analysis revealed that age (OR 1.016, 95% C.I. 1.001–1.032, *P* = 0.037) and ascites (OR 3.505, 95% C.I. 1.358–9.048, *P* = 0.010) were independently associated with the variant type **(**Table 3**)**.

**Table 3:** Extensive evaluations of associations between the clinicopathological variables and the UGT2B28-rs2132039 variant types (i.e. the dependent variable, TT = 0, Non-TT = 1) using univariate and multivariate logistic regression analysis

Characteristic	Odds ratio	95%CI	*P*-value
Univariate analysis			
Gender, male	0.824	0.509–1.334	0.431
Age at diagnosis, years	1.021	1.006–1.036	0.005^*^
Tumor size, cm	0.981	0.932–1.032	0.449
Tumor number	1.084	0.886–1.328	0.432
Capsule	1.094	0.695–1.721	0.699
Tumor grade	0.940	0.698–1.266	0.685
Macrovascular invasion	0.842	0.440–1.610	0.603
Microvascular invasion	0.760	0.493–1.170	0.212
Cirrhosis	1.072	0.713–1.613	0.737
Ascites	3.223	1.264–8.215	0.014^*^
Alcoholism	0.845	0.533–1.340	0.475
HBV	0.710	0.458–1.101	0.126
HCV	1.839	1.148–2.945	0.011^*^
HBV + HCV	1.291	0.644–2.588	0.471
NBNC	0.848	0.479–1.502	0.572
Prothrombin time, second	0.938	0.811–1.084	0.385
AST, U/L	1.002	0.999–1.005	0.246
ALT, U/L	1.003	1.000–1.005	0.095
Bilirubin, mg/dL	0.960	0.808–1.142	0.646
Albumin, g/dL	0.820	0.572–1.175	0.279
Creatinine, mg/dL	0.976	0.790–1.206	0.823
Alpha-fetoprotein, ng/mL	1.000	1.000–1.000	0.377
Recurrent	1.208	0.806–1.811	0.359
Recurrent time, month	0.995	0.988–1.001	0.113
Metastasis	0.692	0.415–1.156	0.160
Metastatic time, month	0.998	0.991–1.004	0.435
Death	0.767	0.416–1.414	0.395
Survival time, month	0.998	0.992–1.005	0.632
Multivariate analysis			
Age, years	1.016	1.001–1.032	0.037^*^
Ascites	3.505	1.358–9.048	0.010^*^
HCV	1.646	0.999–2.713	0.050

*Abbreviations*: *CI* Confidence interval, *HBV* Hepatitis B virus carrier, *HCV* Hepatitis C virus carrier, *HBV + HCV* Co-infection of hepatitis B virus and hepatitis C virus, *NBNC* Non-hepatitis B/hepatitis C virus carrier, *AST* Aspartate aminotransferase, *ALT* Alanine aminotransferase

*P < 0.05 were indicated as bold face with an asterisk

**Fig. 2:**
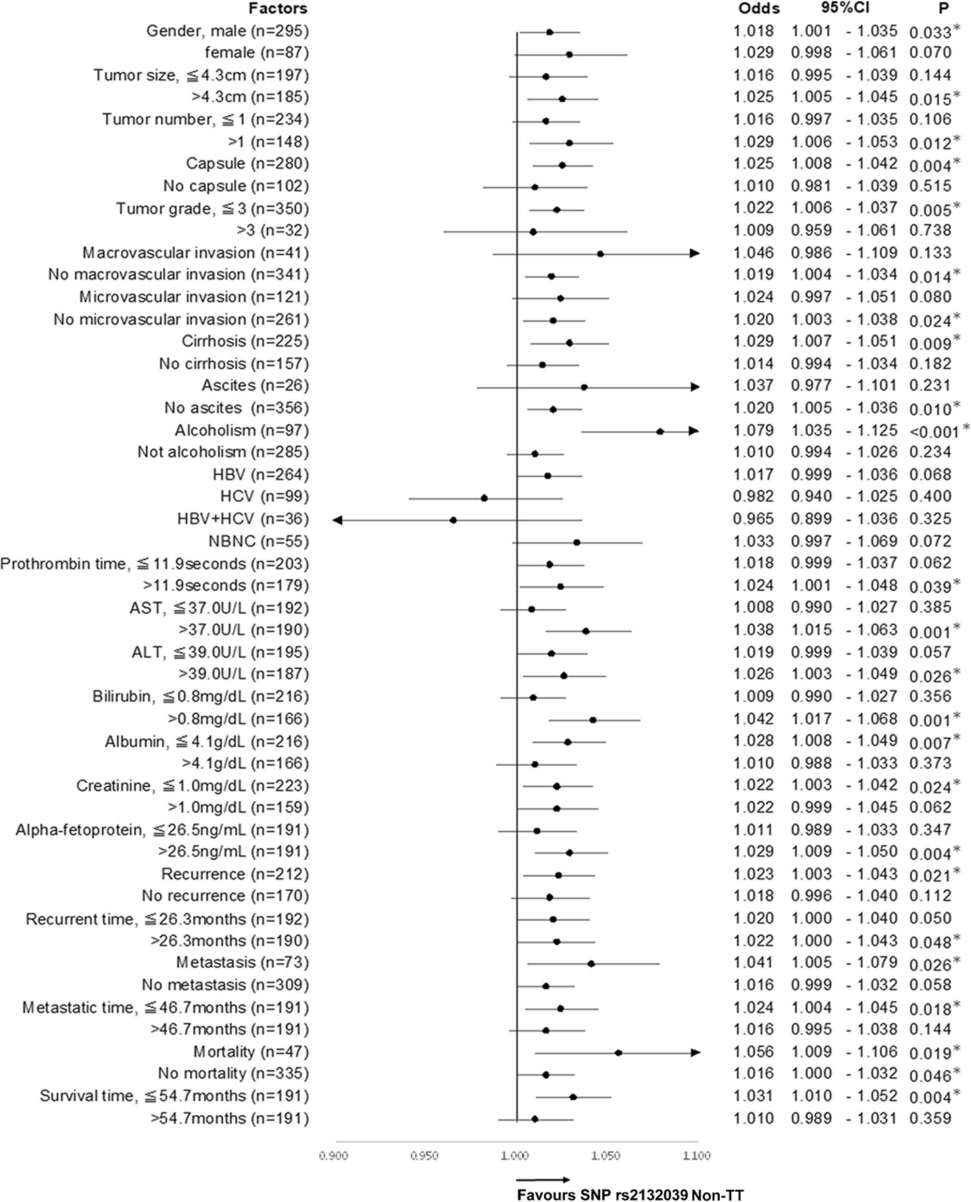
The forest plot of associations between the rs2132039 genomic variant and the age of HCC diagnosis in subgroups of patients stratified by extensive clinicopathological parameters. * indicates a statistically significant association when *P* < 0.05

**Fig. 3:**
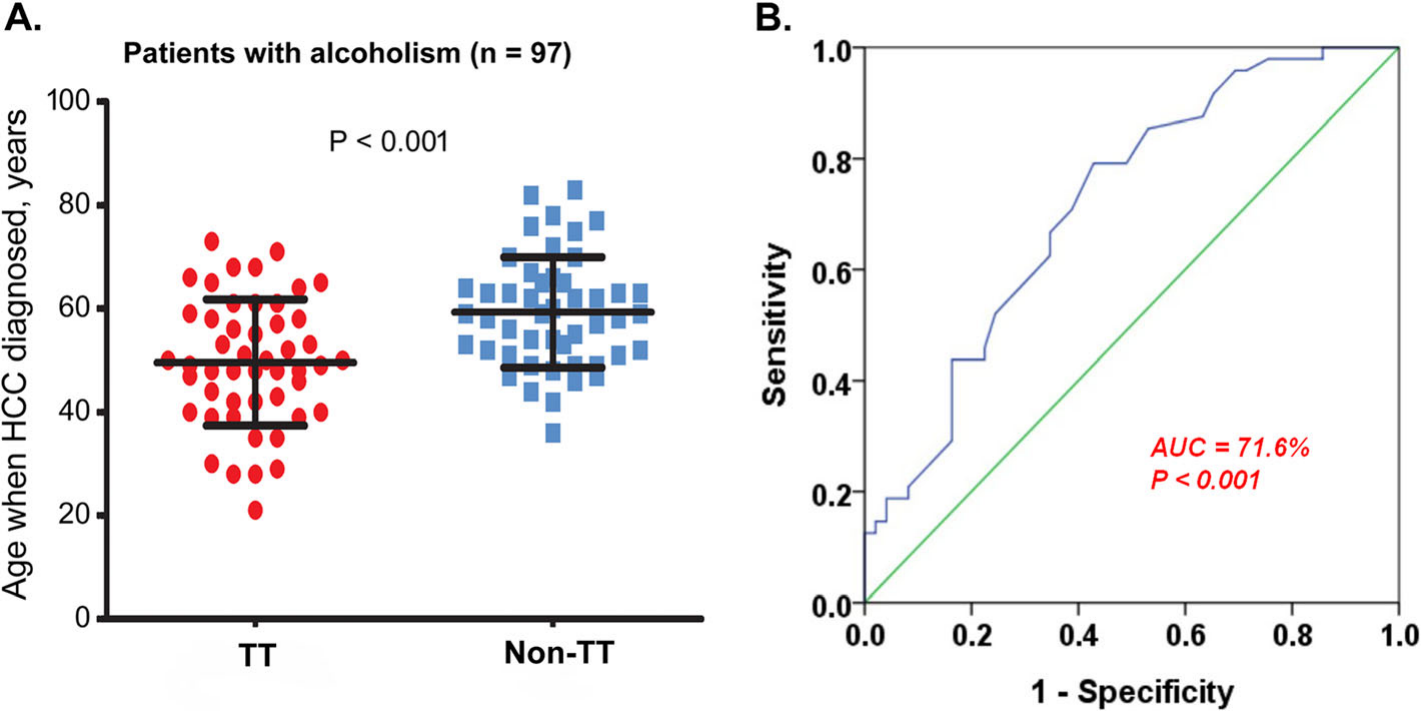
**a** The distribution of age of HCC diagnosis in the subgroup of patients with alcoholism. Red and blue dots represent the patients with rs2132039- “TT” variant type and “Non-TT” variant type respectively. The age of “TT”, 49.6 ± 12.2; the age of “non-TT”, 59.3 ± 10.7 (*P* < 0.001). **b** Receiver operating characteristic curve of the classification of rs2132039 genomic variant using ages. AUC, area under curve

Subgroup analysis was then performed to further evaluate the relationship between the genomic variant and the age. It showed that the genomic variant was most tightly associated with age in alcoholic patients (OR 1.079, 95% C.I. 1.035–1.125, *P* < 0.001) **(**Fig. 2**)**. A large effect size was observed that the age distributions were 59.3 ± 10.7 and 49.6 ± 12.2 years old in patients with “Non-TT” and “TT” variant types, respectively (Fig. 3a). The area under the receiver operating characteristic curve (AUC) was 71.6% when the genomic variant was classified by age (Fig. 3b). Apart from alcoholism, highly significant associations were also found in the subgroups of patients with AST > 37 U/L (OR 1.038, 95% C.I. 1.015–1.063, *P* = 0.001) and bilirubin > 0.8 mg/dL (OR 1.042, 95% C.I. 1.017–1.068, *P* = 0.001, Fig. 2).

Similarly, the variant-ascites and variant-HCV associations in various subgroup was shown in Additional file 2: Figure S2 and Additional file 3: Figure S3.

### *UGT2B28* genomic variant was associated with age of recurrence, metastasis and death in non-censored patients

**Fig. 4:**
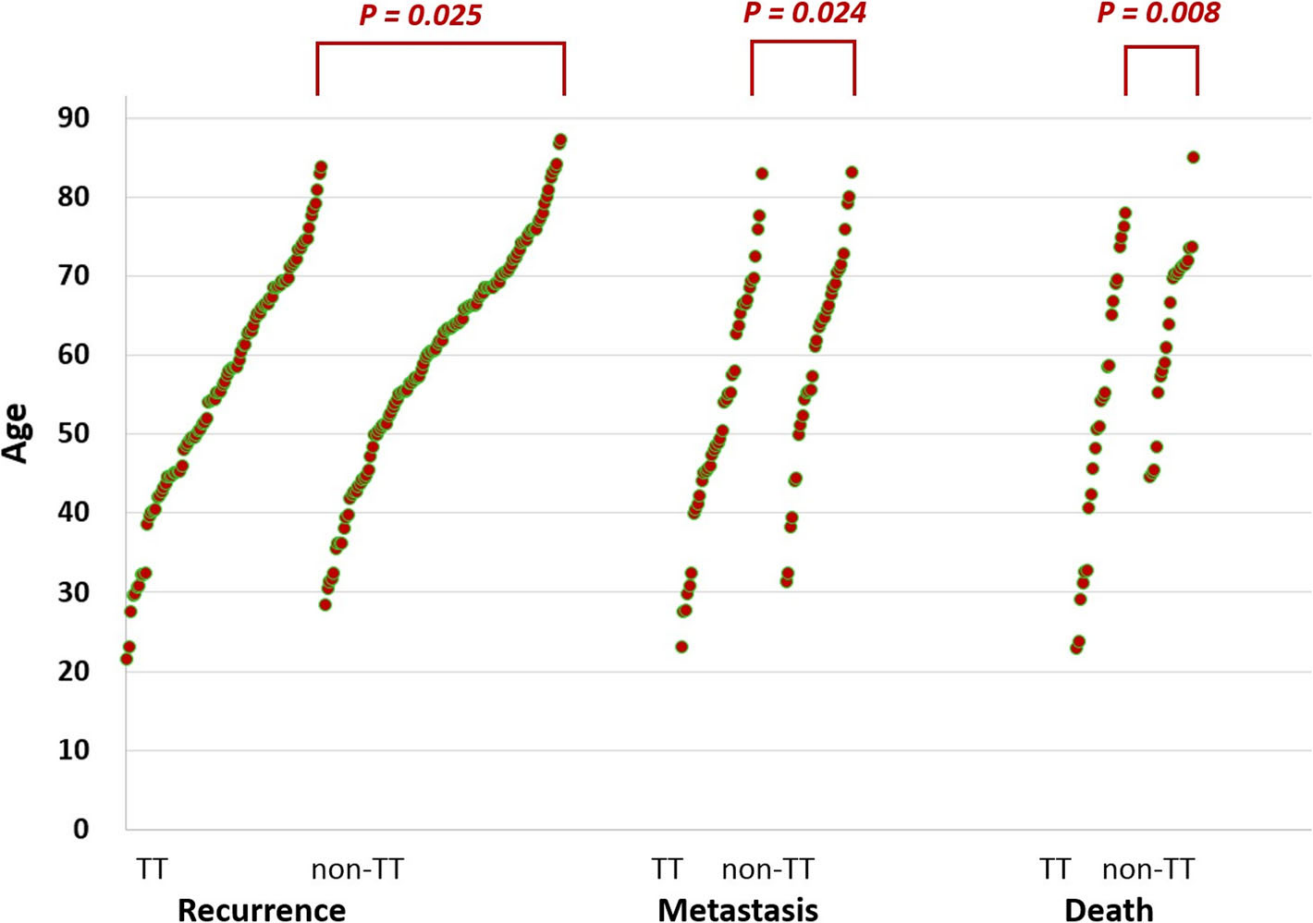
The age distributions of HCC recurrence, metastasis and HCC-related death in patients with different variant types. Two-sample t-test with unequal variances were used

We then analyzed the subsequent clinical events after surgery, including local recurrence, distant metastasis and death in non-censored patients. Patients of the “TT” variant type had a younger age of recurrence (55.8 ± 14.6, *N* = 96) than those of the “Non-TT” variant type (60.3 ± 13.8, *N* = 116, *P* = 0.025, Fig. 4). Also, patients of the “TT” variant type had a younger age of distant metastasis (52.3 ± 15.0, *N* = 40) than those of the “Non-TT” variant type (60.0 ± 13.4, *N* = 33, *P* = 0.024). Among all patients, a total of 47 patients have complete follow-up information until death. Patients of the “TT” variant type had a significantly younger age of HCC-related death (52.2 ± 17.1, *N* = 25) than those of the “Non-TT” variant type (63.7 ± 11.0, *N* = 22, *P* = 0.008).

**Fig. 5:**
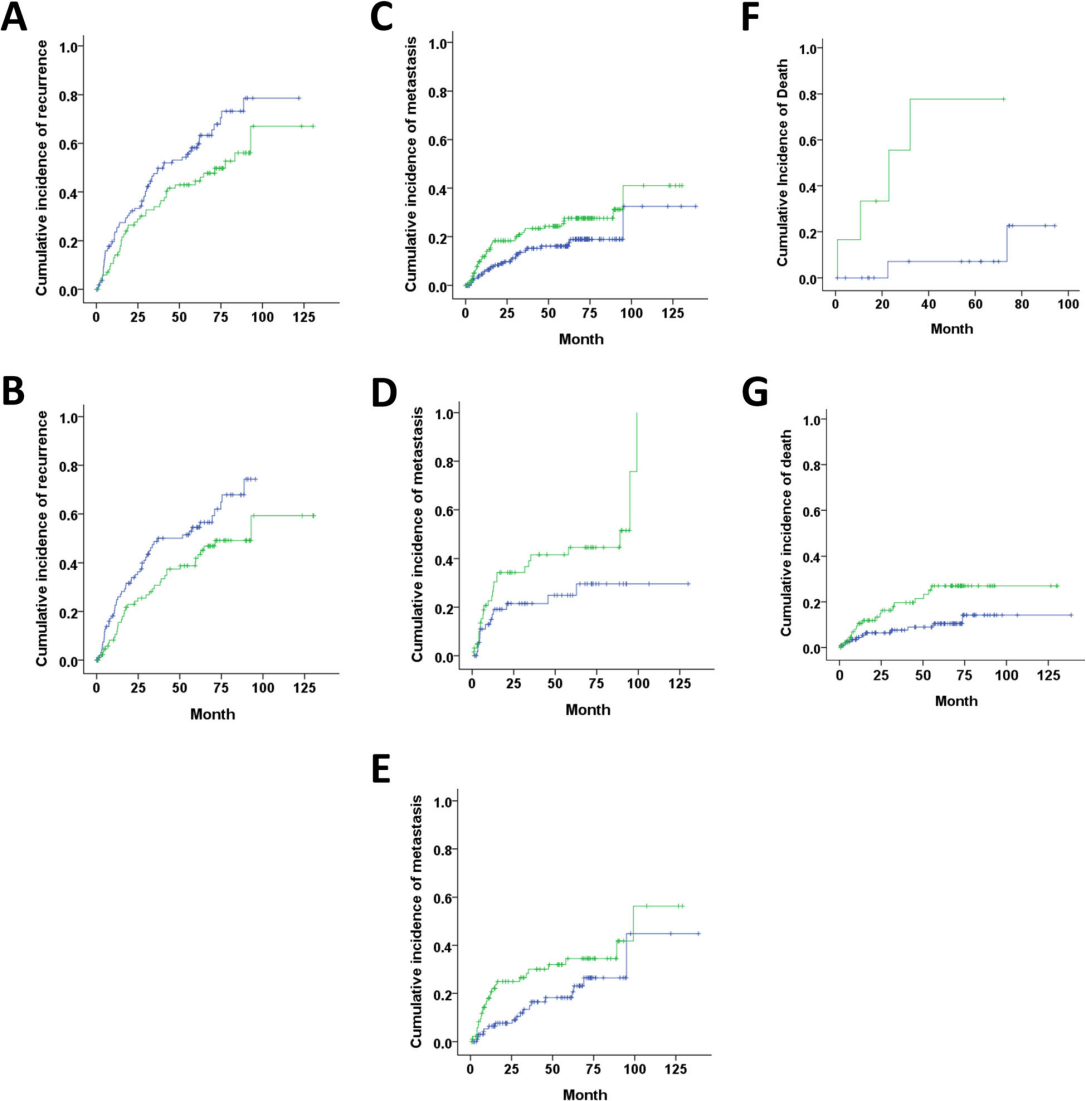
The Kaplan-Meier plots for the cumulative incidence of recurrence in patients of (**a**) smaller tumor size (≦4.3 cm) and (**b**) lower AFP level (≦26.5 ng/mL); for metastasis in patients of (**c**) lower tumor grade (≦3), (**d**) microvascular invasion and (**e**) higher AFP level (> 26.5 ng/mL); for death in patients with (**f**) ascites and (**g**) lower albumin level (≦4.1 g/dL). Green: patients with the variant type “TT”; Blue: patients with the variant type “Non-TT”

We also analyzed censored and non-censored data jointly. The *UGT2B28*-rs2132039-variant type was not associated with post-surgery time to recurrence, time to distant metastasis and death. However, among various subgroups (Additional file 5: Table S2), it was found that the “Non-TT” variant type was associated with higher cumulative incidence of recurrence in the patients with tumor size≦4.3 cm (HR 1.568, 95% CI 1.061–2.315, *P* = 0.024) and AFP≦26.5 ng/mL (HR 1.623; 95% CI 1.067–2.469, *P* = 0.024). The “Non-TT” variant type was associated with lower cumulative incidence of distant metastasis in the patients with tumor grade≦3 (HR 0.599, 95% CI 0.367–0.979, *P* = 0.041), microvascular invasion (HR 0.500, 95% CI 0.257–0.975, *P* = 0.042) and AFP >  26.5 ng/mL (HR 0.538; 95%CI 0.296–0.979, *P* = 0.043). The “Non-TT” variant type was associated with lower cumulative incidence of death in the patients with ascites (HR 0.058, 95% CI 0.006–0.523, *P* = 0.011) and albumin≦4.1 g/dL (HR 0.414; 95% CI 0.197–0.870, *P* = 0.020). The corresponding Kaplan-Meier plots of these subgroups of patients were shown in Fig. 5.

## Discussion

In this study, we only analyzed patients in early HCC stages in the tissue bank, because those in advanced stages were either due to failure of previous treatments (such as surgical resections) or delayed diagnosis. For those with delayed diagnosis, the length of delay was not homogeneous in different people. One major goal of this study was to estimate the age of HCC presentation as early as possible, for the purpose of improving surveillance. The patients with delayed diagnosis may introduce uncontrolled variability to this study. Hence, we screened the tissue bank and identified 382 surgically treated HCC patients whose deposited tissues were available for this study, and other 69 patients whose tissues were no longer available. Clearly, the currently study was limited by the sample availability. A comparison of tumor characteristics showed that most tumor characteristics were similar, except tumor size (Additional file 4: Table S1). The tissue bank supported a wide diversity of clinical investigations, such as the immunohistochemical staining which usually requires tissues with decent sizes [40]. In our data, the tumor size does not associated with the age of presentation (Table 2) and the genomic variant (Table 3). Hence, we assumed the exclusion of the 69 patients did not affect greatly the conclusions of this research.

The investigated genomic biomarker was significantly associated with the ages of HCC presentation, recurrence, distant metastasis and death. We conducted a scrutiny of the post-surgery clinical outcomes. The age disparity of the two variant types showed stronger statistical significance in death ages (*P* = 0.008, *N* = 47) than in recurrence ages (*P* = 0.025, *N* = 212, Fig. 4), despite the sample size was smaller. To explore the genomic effect in detail, we conducted an extensive subgroup analysis and discovered that (1) in the patients of smaller tumor size and lower AFP level, “Non-TT” variant type had higher cumulative incidence of recurrence; (2) in the patients of lower tumor grade, microvascular invasion and higher AFP level, “Non-TT” variant type had lower cumulative incidence of metastasis; (3) in the patients with ascites and lower albumin level (usually advanced cirrhosis), “Non-TT” variant type had lower cumulative incidence of death (Fig. 5). These observations could be generalized that the “Non-TT” variant type indicates better survival, compared with “TT” variant type, in the patients with more advanced HCC (ascites, lower albumin level, microvascular invasion and higher AFP level). The protective effect of “Non-TT” variant in subgroups of patients may contribute toward the more prominent difference in the age distribution of death.

The *UGT2B28* gene encodes a phase-two xenobiotic metabolizing enzyme which can transfer glucuronic acid from uridine diphosphoglucuronic acid to substrates such as bile acids, 5-beta-androstane 3-alpha, 17-beta-diol, estradiol, androsterone, eugenol [30–32], steroid hormones and lipid-soluble drugs [41]. Its role in bile acid metabolism may explain why the association was more prominent in patients with bilirubin > 0.8 mg/dL, and patients with heavy alcohol consumption.

This study was limited by the lack of serum aflatoxin levels, a known liver toxin which has been shown to shorten the time of HCC occurrence in Taiwan and worldwide [42]. Aflatoxin was not routinely measured in patients with chronic liver disease in Taiwan, particularly when the patients already have disease-causing etiology identified such as viral infections and heavy alcohol consumptions. The quantitative aflatoxin measurements were largely missing in the clinical charts and thus were not analyzed.

## Conclusion

Patients with *UGT2B28-*rs2132039 - TT variant type had an earlier presentation of HCC, earlier post-surgery recurrence, metastasis and HCC-related death. The mean age difference of HCC presentation was particularly large (~ 10 years) in alcoholic patients. Such information is helpful for formulating an effective surveillance strategy.

## Supplementary information

**Additional file 1: Figure S1.** The theoretical amplicon sequence based on the human reference genome GRCh38.p7.

**Additional file 2: Figure S2.** The forest plot of odds ratios of the rs2132039 genomic variant with respect to ascites in subgroups of patients stratified by clinicopathological parameters. * indicates a statistically significant association when *P*<0.05.

**Additional file 3: Figure S3.** The forest plot of odds ratios of the rs2132039 genomic variant with respect to HCV infections in subgroups of patients stratified by clinicopathological parameters. * indicates a statistically significant association when *P*<0.05.

**Additional file 4: Table S1.** Tumor characteristics of patients who have deposited their surgical tissues in the tissue bank.

**Additional file 5: Table S2.** The univariate Cox regression analysis of subsequent events after the curative resection in various subgroups (Non-TT = 1, TT = 0).

## Data Availability

De-linked data are available to academic scientists upon request.

## Abbreviations

ALT: Alanine aminotransferase
AST: Aspartate aminotransferase
CI: Confidence interval
HBV: Hepatitis B virus carrier
HCC: Hepatocellular carcinoma
HCV: Hepatitis C virus
HR: Hazard ratio
NBNC: Non-hepatitis B/hepatitis C
