# Supplementary material for: Ages of hepatocellular carcinoma occurrence and life expectancy are associated with a UGT2B28 genomic variation
Source: BMC Cancer. 2019 Dec 5;19:1190. doi: 10.1186/s12885-019-6409-3 (PMC6896495; doi:10.1186/s12885-019-6409-3)
Supplement: Supplementary file 1 — Additional file 1: Figure S1. The theoretical amplicon sequence based on the human reference genome GRCh38.p7. [file 12885_2019_6409_MOESM1_ESM.docx]

**Figure S1.** The theoretical amplicon sequence obtained via the NCBI dbSNP system based on the human reference genome GRCh38.p7.
