# Supplementary material for: Ages of hepatocellular carcinoma occurrence and life expectancy are associated with a UGT2B28 genomic variation
Source: BMC Cancer. 2019 Dec 5;19:1190. doi: 10.1186/s12885-019-6409-3 (PMC6896495; doi:10.1186/s12885-019-6409-3)
Supplement: Supplementary file 2 — Additional file 2: Figure S2. The forest plot of odds ratios of the rs2132039 genomic variant with respect to ascites in subgroups of patients stratified by clinicopathological parameters. * indicates a statistically significant association when P<0.05. [file 12885_2019_6409_MOESM2_ESM.docx]

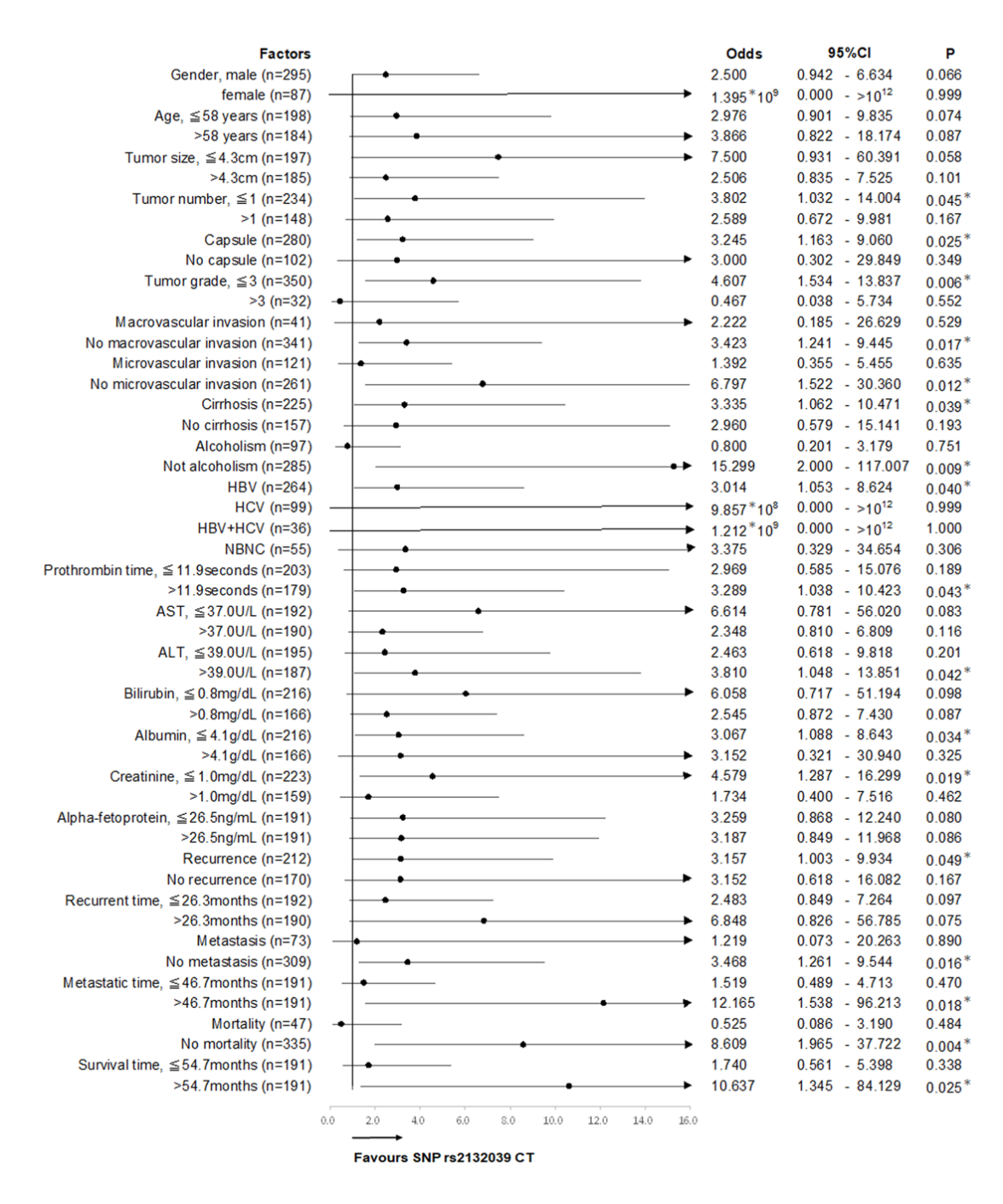


**Figure S2.** The forest plot of odds ratios of the rs2132039 genomic variant with respect to ascites in subgroups of patients stratified by clinicopathological parameters. * indicates a statistically significant association when P<0.05.
