# Supplementary material for: Ages of hepatocellular carcinoma occurrence and life expectancy are associated with a UGT2B28 genomic variation
Source: BMC Cancer. 2019 Dec 5;19:1190. doi: 10.1186/s12885-019-6409-3 (PMC6896495; doi:10.1186/s12885-019-6409-3)
Supplement: Supplementary file 4 — Additional file 4: Table S1. Tumor characteristics of patients who have deposited their surgical tissues in the tissue bank. [file 12885_2019_6409_MOESM4_ESM.docx]

**Table S1**. Tumor characteristics of patients who have deposited their surgical tissues in the tissue bank.

| **Characteristic** | **Patients included** | **Patients excluded** | **P** |
| --- | --- | --- | --- |
| Sample Size | 382 | 69 |  |
| Gender, male, n (%) | 295 (77.2%) | 56 (81.2%) | 0.469 |
| Age at diagnosis, years, median (range) | 58.0 (19.0 - 87.0) | 56.0 (28.0 - 89.0) | 0.304 |
| Tumor number, median (range) | 1.0 (1.0 - 10.0) | 1.0 (1.0 - 5.0) | 0.104 |
| Capsule, n (%) | 280 (73.3%) | 49 (71.0%) | 0.694 |
| Tumor grade, median (range) | 3.0 (1.0 - 4.0) | 3.0 (1.0 - 4.0) | 0.594 |
| Macrovascular invasion, n (%) | 41 (10.7%) | 11 (15.9%) | 0.212 |
| Microvascular invasion, n (%) | 121 (31.7%) | 24 (34.8%) | 0.611 |
| Tumor size > 4.3cm, n (%) | 185 (48.4%) | 47 (68.1%) | **0.003** |
| Cirrhosis, n (%) | 225 (58.9%) | 35 (50.7%) | 0.206 |
