# Supplementary material for: Ages of hepatocellular carcinoma occurrence and life expectancy are associated with a UGT2B28 genomic variation
Source: BMC Cancer. 2019 Dec 5;19:1190. doi: 10.1186/s12885-019-6409-3 (PMC6896495; doi:10.1186/s12885-019-6409-3)
Supplement: Supplementary file 5 — Additional file 5: Table S2. The univariate Cox regression analysis of subsequent events after the curative resection in various subgroups (Non-TT = 1, TT = 0). [file 12885_2019_6409_MOESM5_ESM.docx]

| **Table S2. The univariate Cox regression analysis of subsequent events after the curative resection in various subgroups (Non-TT = 1, TT = 0).** | | | | | | | | | | | | | | | | | | | |
| --- | --- | --- | --- | --- | --- | --- | --- | --- | --- | --- | --- | --- | --- | --- | --- | --- | --- | --- | --- |
| **Parameters** | **Overall survival** | | | | |  |  | **Time to recurrence** | | | | |  | **Time to distant metastasis** | | | | |  |
|  | **SE** | **HR** | **95%CI** | **P-value** |  |  |  | **SE** | **HR** | **95%CI** | **P-value** |  |  | **SE** | **HR** | **95%CI** | **P-value** |  | |
| **All patients (n=382)** | 0.292 | 0.778 | 0.438-1.380 | 0.390 |  |  |  | 0.138 | 1.186 | 0.904-1.555 | 0.218 |  |  | 0.236 | 0.756 | 0.476-1.199 | 0.235 |  | |
| **Gender, male (n=295)** | 0.362 | 0.520 | 0.256-1.057 | 0.071 |  |  |  | 0.156 | 1.195 | 0.880-1.623 | 0.253 |  |  | 0.268 | 0.745 | 0.441-1.259 | 0.271 |  | |
| **female (n=87)** | 0.592 | 2.011 | 0.630-6.417 | 0.238 |  |  |  | 0.302 | 1.224 | 0.677-2.213 | 0.504 |  |  | 0.500 | 0.772 | 0.290-2.059 | 0.605 |  | |
| **Age, ≦58 years** | 0.449 | 0.426 | 0.177-1.027 | 0.057 |  |  |  | 0.192 | 1.086 | 0.745-1.582 | 0.669 |  |  | 0.322 | 0.587 | 0.312-1.105 | 0.099 |  | |
| **>58 years** | 0.438 | 1.391 | 0.589-3.283 | 0.452 |  |  |  | 0.203 | 1.277 | 0.858-1.900 | 0.228 |  |  | 0.367 | 1.100 | 0.536-2.258 | 0.794 |  | |
| **Tumor size, ≦4.3cm (n=197)** | 0.541 | 1.153 | 0.399-3.330 | 0.792 |  |  |  | 0.199 | 1.568 | 1.061-2.315 | **0.024^＊^** |  |  | 0.388 | 0.975 | 0.456-2.083 | 0.947 |  | |
| **>4.3cm (n=185)** | 0.353 | 0.691 | 0.346-1.379 | 0.294 |  |  |  | 0.197 | 0.934 | 0.635-1.373 | 0.728 |  |  | 0.301 | 0.687 | 0.381-1.238 | 0.211 |  | |
| **Tumor number, ≦1 (n=234)** | 0.372 | 1.041 | 0.502-2.157 | 0.915 |  |  |  | 0.180 | 1.183 | 0.831-1.684 | 0.352 |  |  | 0.321 | 0.959 | 0.511-1.798 | 0.895 |  | |
| **>1 (n=148)** | 0.484 | 0.469 | 0.182-1.211 | 0.118 |  |  |  | 0.217 | 1.114 | 0.729-1.703 | 0.618 |  |  | 0.350 | 0.534 | 0.269-1.060 | 0.073 |  | |
| **Capsule (n=280)** | 0.350 | 0.695 | 0.350-1.380 | 0.299 |  |  |  | 0.160 | 1.110 | 0.811-1.518 | 0.515 |  |  | 0.291 | 0.676 | 0.382-1.197 | 0.179 |  | |
| **No capsule (n=102)** | 0.537 | 0.991 | 0.346-2.838 | 0.987 |  |  |  | 0.277 | 1.465 | 0.850-2.523 | 0.169 |  |  | 0.402 | 0.990 | 0.450-2.179 | 0.981 |  | |
| **Tumor grade, ≦3 (n=350)** | 0.305 | 0.657 | 0.362-1.194 | 0.168 |  |  |  | 0.145 | 1.180 | 0.888-1.567 | 0.253 |  |  | 0.251 | 0.599 | 0.367-0.979 | **0.041^＊^** |  | |
| **>3 (n=32)** | 4.743 | 70.674 | 0.006-770703.969 | 0.369 |  |  |  | 0.474 | 1.192 | 0.470-3.020 | 0.711 |  |  | 3.399 | 86.626 | 0.111-67686.295 | 0.189 |  | |
| **Macrovascular invasion (n=41)** | 0.731 | 1.724 | 0.411-7.228 | 0.456 |  |  |  | 0.399 | 0.742 | 0.339-1.621 | 0.454 |  |  | 0.536 | 1.074 | 0.376-3.070 | 0.894 |  | |
| **No macrovascular invasion (n=341)** | 0.323 | 0.670 | 0.355-1.261 | 0.214 |  |  |  | 0.148 | 1.269 | 0.949-1.697 | 0.108 |  |  | 0.265 | 0.730 | 0.435-1.227 | 0.235 |  | |
| **Microvascular invasion (n=121)** | 0.443 | 0.551 | 0.231-1.315 | 0.179 |  |  |  | 0.214 | 1.179 | 0.774-1.794 | 0.443 |  |  | 0.341 | 0.500 | 0.257-0.975 | **0.042^＊^** |  | |
| **No microvascular invasion (n=261)** | 0.403 | 1.107 | 0.502-2.440 | 0.801 |  |  |  | 0.183 | 1.301 | 0.909-1.860 | 0.150 |  |  | 0.349 | 1.257 | 0.634-2.493 | 0.512 |  | |
| **Cirrhosis (n=225)** | 0.388 | 1.099 | 0.514-2.351 | 0.807 |  |  |  | 0.178 | 1.406 | 0.991-1.994 | 0.056 |  |  | 0.322 | 0.894 | 0.475-1.681 | 0.728 |  | |
| **No cirrhosis (n=157)** | 0.469 | 0.489 | 0.195-1.226 | 0.127 |  |  |  | 0.224 | 0.974 | 0.628-1.510 | 0.905 |  |  | 0.349 | 0.631 | 0.318-1.250 | 0.187 |  | |
| **Ascites (n=26)** | 1.122 | 0.058 | 0.006-0.523 | **0.011^＊^** |  |  |  | 0.579 | 0.699 | 0.224-2.175 | 0.536 |  |  | 1.414 | 0.282 | 0.018-4.504 | 0.370 |  | |
| **No ascites (n=356)** | 0.313 | 0.910 | 0.493-1.679 | 0.763 |  |  |  | 0.144 | 1.173 | 0.884-1.556 | 0.268 |  |  | 0.239 | 0.816 | 0.511-1.304 | 0.395 |  | |
| **Alcoholism (n=97)** | 0.571 | 0.549 | 0.179-1.680 | 0.293 |  |  |  | 0.267 | 1.269 | 0.752-2.144 | 0.373 |  |  | 0.445 | 0.921 | 0.385-2.203 | 0.853 |  | |
| **Not alcoholism (n=285)** | 0.343 | 0.889 | 0.454-1.743 | 0.733 |  |  |  | 0.162 | 1.161 | 0.846-1.595 | 0.355 |  |  | 0.279 | 0.704 | 0.407-1.217 | 0.209 |  | |
| **HBV (n=264)** | 0.369 | 0.597 | 0.290-1.229 | 0.161 |  |  |  | 0.166 | 1.150 | 0.830-1.593 | 0.402 |  |  | 0.289 | 0.667 | 0.378-1.175 | 0.161 |  | |
| **HCV (n=99)** | 0.807 | 2.363 | 0.486-11.502 | 0.287 |  |  |  | 0.292 | 1.349 | 0.761-2.392 | 0.305 |  |  | 0.611 | 1.817 | 0.549-6.012 | 0.328 |  | |
| **HBV+HCV (n=36)** | 1.225 | 0.283 | 0.026-3.120 | 0.302 |  |  |  | 0.519 | 1.567 | 0.566-4.336 | 0.387 |  |  | 0.893 | 1.634 | 0.284-9.406 | 0.583 |  | |
| **NBNC (n=55)** | 0.646 | 0.704 | 0.198-2.497 | 0.586 |  |  |  | 0.374 | 1.200 | 0.577-2.498 | 0.626 |  |  | 0.518 | 0.895 | 0.324-2.470 | 0.830 |  | |
| **Prothrombin time,≦11.9seconds(n=203)** | 0.401 | 0.797 | 0.364-1.748 | 0.572 |  |  |  | 0.199 | 1.092 | 0.740-1.614 | 0.657 |  |  | 0.321 | 0.655 | 0.349-1.228 | 0.187 |  | |
| **>11.9seconds (n=179)** | 0.428 | 0.740 | 0.320-1.712 | 0.481 |  |  |  | 0.194 | 1.319 | 0.902-1.929 | 0.153 |  |  | 0.349 | 0.884 | 0.446-1.752 | 0.724 |  | |
| **AST, ≦37.0U/L (n=192)** | 0.494 | 0.438 | 0.166-1.152 | 0.094 |  |  |  | 0.211 | 1.264 | 0.836-1.911 | 0.266 |  |  | 0.352 | 0.721 | 0.361-1.438 | 0.353 |  | |
| **>37.0U/L (n=190)** | 0.383 | 1.077 | 0.509-2.279 | 0.847 |  |  |  | 0.183 | 1.063 | 0.742-1.523 | 0.738 |  |  | 0.319 | 0.771 | 0.413-1.440 | 0.414 |  | |
| **ALT, ≦39.0U/L (n=195)** | 0.403 | 0.573 | 0.260-1.262 | 0.167 |  |  |  | 0.204 | 1.306 | 0.876-1.946 | 0.190 |  |  | 0.317 | 0.673 | 0.361-1.254 | 0.212 |  | |
| **>39.0U/L (n=187)** | 0.442 | 1.137 | 0.478-2.703 | 0.771 |  |  |  | 0.189 | 1.073 | 0.741-1.554 | 0.709 |  |  | 0.357 | 0.930 | 0.461-1.873 | 0.838 |  | |
| **Bilirubin, ≦0.8mg/dL (n=216)** | 0.428 | 0.819 | 0.354-1.898 | 0.642 |  |  |  | 0.181 | 1.222 | 0.857-1.743 | 0.268 |  |  | 0.296 | 0.838 | 0.469-1.498 | 0.551 |  | |
| **>0.8mg/dL (n=166)** | 0.400 | 0.706 | 0.322-1.547 | 0.384 |  |  |  | 0.216 | 1.146 | 0.751-1.749 | 0.528 |  |  | 0.388 | 0.617 | 0.288-1.321 | 0.214 |  | |
| **Albumin, ≦4.1g/dL (n=216)** | 0.379 | 0.414 | 0.197-0.870 | **0.020^＊^** |  |  |  | 0.183 | 0.976 | 0.681-1.397 | 0.893 |  |  | 0.299 | 0.622 | 0.346-1.119 | 0.113 |  | |
| **>4.1g/dL (n=166)** | 0.508 | 2.074 | 0.766-5.618 | 0.151 |  |  |  | 0.213 | 1.437 | 0.946-2.181 | 0.089 |  |  | 0.384 | 0.918 | 0.433-1.948 | 0.824 |  | |
| **Creatinine, ≦1.0mg/dL (n=223)** | 0.364 | 0.539 | 0.264-1.101 | 0.090 |  |  |  | 0.180 | 1.136 | 0.798-1.618 | 0.479 |  |  | 0.290 | 0.586 | 0.332-1.035 | 0.065 |  | |
| **>1.0mg/dL (n=159)** | 0.505 | 1.453 | 0.541-3.906 | 0.459 |  |  |  | 0.217 | 1.262 | 0.825-1.930 | 0.283 |  |  | 0.409 | 1.177 | 0.528-2.624 | 0.690 |  | |
| **Alpha-fetoprotein,≦26.5ng/mL (n=191)** | 0.460 | 1.079 | 0.438-2.659 | 0.868 |  |  |  | 0.214 | 1.623 | 1.067-2.469 | **0.024^＊^** |  |  | 0.380 | 1.173 | 0.557-2.470 | 0.675 |  | |
| **>26.5ng/mL (n=191)** | 0.382 | 0.579 | 0.274-1.225 | 0.153 |  |  |  | 0.182 | 0.896 | 0.627-1.281 | 0.548 |  |  | 0.305 | 0.538 | 0.296-0.979 | **0.043^＊^** |  | |

*P<0.05

Abbreviations: SE, standard error; HR, hazard ratio; CI, confidence interval; HBV, hepatitis B virus carrier; HCV, hepatitis C virus carrier; HBV+HCV, co-infection of hepatitis B virus and hepatitis C virus; NBNC, non-hepatitis B/hepatitis C virus carrier; AST, aspartate aminotransferase; ALT, alanine aminotransferase
